# It begins with the right questions

**DOI:** 10.1308/rcsann.2026.0091

**Published:** 2026-09-01

**Authors:** K Mahawar

**Affiliations:** *Annals* Editor-in-Chief

**Figure:**
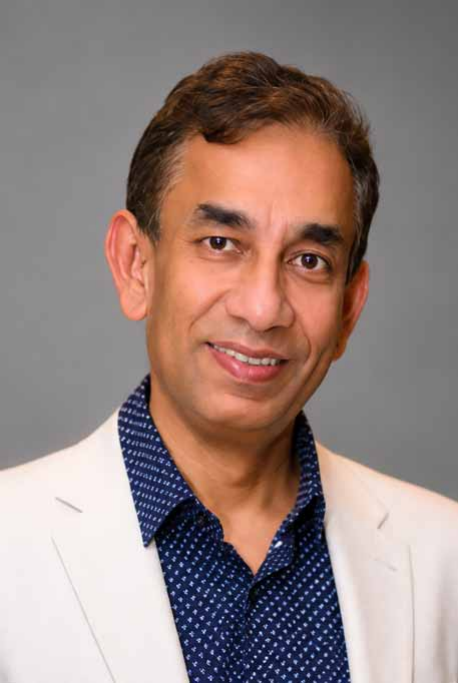


Taking on the role of editor-in-chief of one of the world’s most distinguished surgical journals is both a privilege and a profound responsibility. It also prompts an inevitable question: how does one build on a legacy shaped by generations of editors, authors, reviewers and readers? I see myself simply as a custodian of that legacy.

I could use this inaugural editorial to outline an ambitious programme for the journal. I could explain how I hope to improve the impact factor (which, at 2.0, is healthy by any measure) or how we might enhance our altmetric performance and international profile. I could draw comparisons with journals that rank more highly and present a list of initiatives designed to close the gap.

But that would assume I already know the answers. More importantly, it would imply that those who came before me had somehow overlooked them. Nothing could be further from the truth. I have the good fortune of succeeding a distinguished line of editors-in-chief, each of whom has strengthened the journal in their own way. I inherit not a journal in need of rescue but one in excellent health.

The real challenge, therefore, is not to begin with answers but with questions. What is the primary purpose of this journal? Is it to advance surgical science, improve everyday surgical practice, or both? How do we balance methodological excellence with clinical relevance? Should we broaden our international reach while remaining true to our roots? How can we work more closely with specialist societies, training bodies and researchers? Should we see our role as extending beyond publishing research to helping develop the research capability that underpins it? And how do we better recognise the reviewers, whose expertise and generosity sustain scientific publishing, and yet too often go unnoticed?

These are not questions for the editor-in-chief to answer alone. They belong to all of us: the President of the Royal College of Surgeons of England (who himself once served in this role), the Council, the publishing team, and our editors, reviewers, authors and readers. I hope that many of you have already contributed your thoughts through the recent fellows and members consultation. If not, I warmly invite you to do so (https://ow.ly/FzcK50ZfNOE). A scientific journal is, above all, a collective endeavour.

Before looking ahead, it is only right to acknowledge my predecessor, Professor Benedict Rogers. Over six years of dedicated leadership, he has strengthened the journal and leaves it in an enviable position. It is a privilege to build on the foundations he has laid.

I am particularly delighted to assume this role at a landmark moment in the journal’s history. Next year, we celebrate our 80^th^ anniversary. Coincidentally, India, the country of my birth, will also mark 80 years of independence. If that still does not quite convey the passage of time, consider that London Heathrow Airport celebrates its 80^th^ anniversary this year.

The years ahead will undoubtedly bring new challenges to surgical publishing. There will be new technologies, changing expectations and different ways of measuring success. Nevertheless, I hope that one principle will remain constant: if we continue to ask the right questions, we stand the best chance of finding the right answers.

